# An improved synthesis of a cyclopropene-based molecule for the fabrication of bioengineered tissues via copper-free click chemistry

**DOI:** 10.1177/2280800019844746

**Published:** 2019-06-21

**Authors:** Marco M Meloni, Stephen Barton, Juan C Kaski, Wenhui Song, Taigang He

**Affiliations:** 1 The Cardiology Academy Group, St George’s University of London, London, UK; 2 School of Pharmacy and Chemistry, Kingston University, London, UK; 3 UCL Centre for Biomaterials, University College London, London, UK; 4 Royal Brompton Hospital, Imperial College London, London, UK

**Keywords:** Biomaterials, copper-free click chemistry, methyl cyclopropenes, synthesis, vascular grafts, contrast agent

## Abstract

**Background:**

Since its introduction in the field of biological imaging, the use of copper-free click chemistry has been extended to produce improved materials for vascular surgery, ophthalmology, environmental, and automotive applications. This wide applicability suggests that larger quantities of the chemical reagents for copper-free click chemistry will be required in the future. However, the large-scale synthesis of such chemicals has been barely investigated. A possible reason is the shortage of reliable synthetic protocols to obtain large quantities of these building blocks. We therefore present in this paper an improved synthetic protocol to obtain a cyclopropene-based carbonate, a key building block for the well-known copper-free click chemistry.

**Method:**

Our protocol builds upon an already available method to obtain a cyclopropene-based carbonate. When scaled up, several parameters of this method were changed in order to obtain an improved yield. First, the use of lower temperatures and slower addition rates of the chemicals avoided the formation of detrimental hotspots in the reaction system. Second, the use of less hygroscopic solvents minimized the decomposition of the cyclopropene carbonate. Finally, chromatographic purifications were minimized and improved by using deactivated silica.

**Results:**

We obtained the compound (2-methylcycloprop-2-en-1-yl)methyl (4-nitrophenyl) carbonate, a key building block for copper-free click chemistry, in an unprecedented 60% overall yield on a six-gram scale.

**Conclusions:**

Our improved synthetic protocol demonstrates the potential of large-scale production of improved materials using click chemistry, with potential future applications in the fields of molecular imaging, vascular surgery, ophthalmology, and theranostics.

## Introduction

The growing demand for theranostics for various diseases has stimulated the development of novel materials capable of integrating diagnostic and therapeutic entities within a single formulation.^1–3^ Few developed biomaterials can currently improve both imaging contrast and delivery efficacy and mimic the native extracellular matrix (ECM) of a tissue. To address these needs, drug carriers or synthetic scaffolds are often required for surface biofunctionalization in order to promote cell attachment, growth, and differentiation.^4^ Several biodegradable polymers such as polycaprolactone (PCL) and polylactic acid (PLA) have been already fabricated as micro/nanoparticles for drug encapsulation and tissue engineering. Unfortunately, their low drug loading and targeting efficacy, poor biofunction, and mismatched mechanical properties have narrowed their medical applications in tissue repair and surgical reconstruction.^2^ Keeping the correct balance between mechanical strength, cytotoxicity, easy biofunctionalization, and controllable biodegradation rate of a biomaterial remains a current challenge.

This challenge has been partially addressed by modifying the matrix of the biomaterial with specific chemical groups like amides, urethanes, or ureas, affording materials with improved mechanical strength and/or biofunctionalization.^5–7^ However, such chemical groups can negatively affect the biodegradation rate and cytotoxicity of the polymer. The synthetic protocols required to introduce these chemical groups can also generate toxic byproducts,^8,9^ hampering translation, large-scale synthesis, and commercialization of such biomaterials into the clinical market.

Copper-free click chemistry has recently become a promising tool for the synthesis and functionalization of materials.^10,11^ Click reactions are broad in scope, simple in operation, and easy to carry out at room or biological temperature (25 or 37°C). They eliminate the use of cytotoxic copper catalysts and are inert to both water and oxygen. They can generate minimal, inoffensive byproducts and provide the best yield with the highest reaction rates. Originally developed to image biomolecules in living systems,^12^ copper-free click chemistry gradually emerged as a multipurpose toolbox to prepare a broad range of biocompatible materials. Examples are vascular grafts and hydrogels for intraocular lenses,^13,14^ nanocomposites for water purification, and self-repairing and fire-retardant polymers with improved resistance to mechanical and thermal stress.^15,16^ The biomaterials produced have displayed improved performance both *in vitro* and *in vivo*. An example is the fabrication of vascular scaffolds based on a poly-1,8-azide-based octanediol citrate elastomer (POC-azide scaffolds), which enabled facile bioconjugation of heat-labile biomolecules via copper-free click reaction in aqueous environment at room temperature. Peptidomimetics which promote adhesion and proliferation of endothelial cells were exemplarily clicked onto POC-azide scaffolds, affording vascular grafts with much improved mechanical strength, up to 40 MPa of tensile stress,^14,16,17^ in a sheep model.^18^ It is therefore anticipated that copper-free click chemistry will address the growing demand for improved materials for clinical imaging, surgery, drug delivery, and environmental chemistry.

**Scheme 1.**
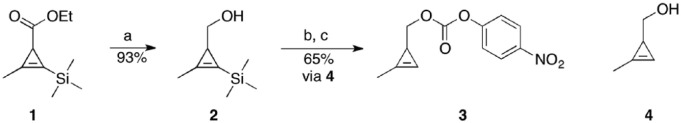
(a) Diisobutylaluminum hydride (DIBAL-H), THF, −10°C to 25°C. (b) *n-*Tetrabutylammonium fluoride (TBAF), CH_2_Cl_2_, 25°C. (c) *p*-Nitrophenyl (PNP) chloroformate, pyridine, CH_2_Cl_2_, 0°C to 25°C.

Albeit promising, the use of copper-free click chemistry for the large-scale preparation of promising engineered and biomedical materials remains largely unexplored. A possible reason is a lack of synthetic protocols to obtain a large amount of the building blocks required for this type of chemistry. The identification of better methods or improvement of existing protocols can accelerate the large-scale production and the marketing of promising engineered and biomedical materials. In this paper we report an optimized and efficient protocol to obtain a cyclopropene carbonate molecule **3** (Scheme 1), a building block required to perform the inverse electron demand Diels–Alder (IEDDA) reaction. This reaction stands out from other biorthogonal reactions for its unmatchable kinetics, excellent orthogonality, and biocompatibility and has already gained wide applications in chemical biology.^19^

## Materials and methods

### Chemicals and solvents

Chemicals and anhydrous solvents were purchased from commercial sources (Sigma Aldrich, Acros, and Alfa Aesar) and used without further purification.

### Glassware

All the experiments were performed in oven-dried glassware (80°C) and under nitrogen atmosphere.

### Chromatography

Flash chromatography was performed on deactivated silica gel (Merck 60 Å, 230–400 Mesh). Thin layer chromatography (TLC) was performed on Merck glass-backed plates pre-coated with silica (0.2 mm, F254). The presence of desired compounds on TLC plates was confirmed either by ultraviolet fluorescence (λ = 254 nm) or by development with a KMnO_4_ solution.

### Nuclear magnetic resonance (NMR) analysis

NMR spectra were recorded at 400 MHz on a Bruker AV-400 instrument. Chemical shifts (δ) are quoted in parts per million (ppm).

### Preparation of deactivated silica gel

A round-bottomed flask was charged with a solution of a CH_2_Cl_2_ containing 5% Et_3_N (1 L). Flash silica gel (500 g) was then added portion-wise under vigorous stirring. The flask was sealed and the suspension was further stirred for 24 h. The silica gel was then filtered on a Buchner funnel, washed twice with CH_2_Cl_2_ (3× 400 mL), and dried under high vacuum at 60°C.

### Preparation and characterization of (2-methyl-3-(trimethylsilyl) cycloprop-2-en-1-yl) methanol (**2**) from ester (**1**)

**Scheme 2.**
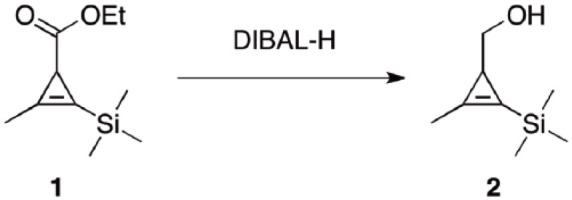
Preparation of alcohol **2**. R_f_ = 0.20 (petrol:Et_2_O 17:3). ^1^H NMR (CDCl_3_, 400 MHz): δ = 3.49 (d, *J* = 4.0 Hz, 2H), 2.21 (s, 3H), 1.56 (t, *J* = 4.0 Hz, 1H), 1.01 (br s, 1H), 0.17 (s, 9H). The ^1^H NMR data reported are in agreement with previous reports for this compound in the literature.^21^

As shown in Scheme 2, a three-neck, round-bottomed flask was equipped with ethyl 2-methyl-3-(trimethylsilyl) cycloprop-2-enecarboxylate **1** (8.1 g, 41 mmol), an internal thermometer, and tetrahydrofuran (THF, 50 mL). The resulting solution was stirred and cooled at −10°C. DIBAL-H (82 mL, 82 mmol, 1M in THF) was added via syringe pump over a period of 1 h and the system gradually warmed to 0°C. TLC (Petrol Et_2_O 17:3) showed starting material left. Additional DIBAL-H (62 mL, 62 mmol, 1M in THF) was added over 15 min, the system warmed to 5°C and stirred for an additional hour. Upon disappearance of the starting material (TLC Petrol Et_2_O 17:3) the system was cooled at 0°C and saturated Rochelle’s salt was added dropwise until gas evolution ceased (**CAUTION**: after an initial induction period of 1–2 min a highly exothermal and bubbling reaction was observed). The pink gel was vigorously stirred and extracted with Et_2_O (4× 200 mL). The organics were combined, washed with brine (4× 300 mL) and dried (Na_2_SO_4_). Removal of the solvent under reduced pressure afforded alcohol **2** as a clear oil (5.9 g, 93%). No further purification was required.

### Preparation and characterization of (2-methylcycloprop-2-en-1-yl) methyl (4-nitrophenyl) carbonate (**3**) from alcohol (**2**)

**Scheme 3.**
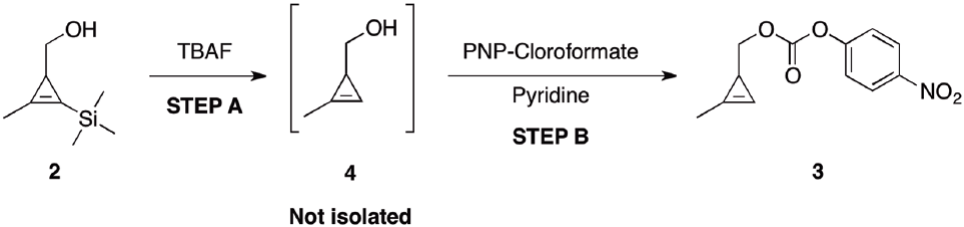
Preparation of carbonate **3**. R_f_ = 0.60 (hexanes Et_2_O 9:1). ^1^H NMR (CDCl_3_, 400 MHz) δ = 8.27 (d, *J* = 11.0 Hz, 2H), 7.38 (d, *J* = 11.0 Hz, 2H), 6.61 (s, 1H), 4.20 (dd, *J* = 12.0, 8.0, Hz, 1H), 4.14 (dd, *J* = 12.0, 8.0 Hz, 1H), 2.17 (d, *J* = 4.0 Hz, 3H), 1.78 (td, *J* = 8.0, 4.0 Hz, 1H). The ^1^H NMR data reported were in agreement with those previously reported in the literature.^24^

As shown in Scheme 3 (step A), a one-neck, round-bottomed flask was charged with (2-methyl-3-(trimethylsilyl) cycloprop-2-en-1-yl)methanol **2** (5.9 g, 38 mmol) and CH_2_Cl_2_ (150 mL). TBAF (38 ml, 1M in THF) was then slowly added via a dropping funnel over 20 min. After 40 min, TLC analysis of the reaction mixture (hexanes:ether 1:1) showed complete disappearance of the starting material (R_f_ = 0.57) and the formation of a single, more polar compound (R_f_ = 0.37). The organics were then washed with brine (3× 50 mL), collected and vigorously stirred over sodium sulfate for 3 h. The organics were then filtered and immediately used in the next step. In step B, a two-necked flask was charged with the organics from step A. Additional CH_2_Cl_2_ (100 mL) and pyridine (7.7 mL, 95 mmol compared to **2**) were added and the system cooled at 0 °C (internal thermometer used). A solution of PNP chloroformate (15.3 g, 76 mmols compared to **2**) in CH_2_Cl_2_ (100 mL) was slowly added over 1 h under vigorous stirring. The solution was gradually warmed to 25°C and stirred for an additional 24 h. The reaction mixture was partitioned between Et_2_O (400 mL) and 1M HCl (200 mL). The organics were further washed with saturated NaHCO_3_ (2× 200 mL), brine (200 mL), and dried (Na_2_SO_4_). Removal of the solvent under reduced pressure (temperature not exceeding 35°C) and chromatography on deactivated silica (hexanes:Et_2_O 9:1) afforded carbonate **3** as a colorless oil (6.2 g, 65% overall yield over two steps starting from alcohol **2** and 60% overall yield from ester **1**).

## Results and discussion

**Scheme 4.**
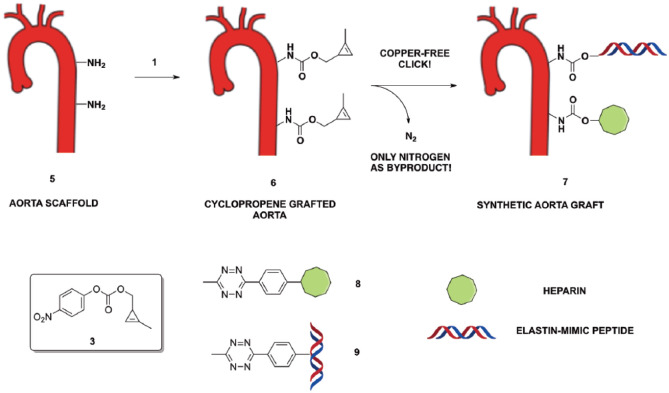
Schematic illustration of biofunctionalization approach to a synthetic aorta graft.

Cyclopropene-based carbonate **3** (Scheme 1) is an efficient molecule for functionalization of micro/nanoparticles and scaffold/implants owing to its high reactivity of **3** with amines.^20,24^ Scheme 4 proposes a potential approach to preparation of a cyclopropene-coated aorta scaffold or graft **6**. This latter can be further functionalized with a tetrazine-based heparin (**8**), a collagen mimic peptide or other protein (**9**) using the IEDDA reaction. It is envisaged that this approach will be straightforward and versatile to achieve a fully biocompatible aorta graft (**7**) for several reasons. First, published data showed that heparin confers anti-thrombogenic properties of the tissue.^21^ Second, collagen-mimic peptides can allow endothelial cell adhesion and proliferation on the tissue surface.^22^ Third, bioconjugation via IEDDA occurs quickly at 25°C and generates non-toxic nitrogen as the only byproduct. This approach could therefore afford many other medical applications, such as targeted imaging contrast agents and drug delivery.

Given the potential for a wide range of applications, a scale-up production of a large amount (5–10 g) of cyclopropene-based carbonate **3** is required. After an initial success in obtaining **3** on a small quantity similar to that reported in the literature (200 mg),^19,23^ a pilot study to scale up by using the same overall methodology (Scheme 1) was tried without success. Only 10% yield of carbonate **3** was obtained. Several possible reasons for such a low yield were considered. First, non-uniform temperature distribution generated the presence of local hotspots during the scaled-up synthesis of both **2** and **3**, potentially affecting the overall performance of the process.^24,25^ As a proof, a rapid temperature increase of the system was observed when preparing intermediates **2** and **3** on a large scale. Second, the use of a hygroscopic solvent like THF in the last stage of the synthesis could have caused a partial loss of intermediate **4** upon aqueous workup.^24^ Third, the potential presence of water in the THF could have promoted an *in-situ* hydrolysis of **3**. Finally, the partial instability of **3** (demonstrated by TLC) towards silica could have decreased its recovery after its chromatography purification. Based on these hypotheses, effective modifications were introduced to the overall synthetic protocol, with the aim to improve the overall yield of carbonate **3** and facilitate the functionalization of a fully biocompatible aorta graft.

The improved approach started with ester **1**, which was obtained on a 10-gram scale using a literature method. ^21^ We then moved to reduce **1** to alcohol **2** with DIBAL-H on a large scale. As originally reported, a solution of ester **1** was added to a precooled solution of DIBAL-H at 4°C over 1 min.^21^ Upon scale-up, this approach resulted in a lack of temperature control of the system and after workup and chromatography, alcohol **2** was obtained in a lower yield (50%) (Table 1), compared to what was previously reported (71%).^21^ To avoid sudden rise in reaction temperature, the DIBAL-H solution was added much more slowly (over 1 h) to a pre-cooled solution of ester **1**, which was kept this time at −10°C. These modifications did not cause any rise in the temperature, indicating the absence of local hot spots in the system. After workup, the yield of alcohol **2** (93%) was significantly improved with a purity greater than 95% by TLC and NMR analysis. ^1^H NMR data of **2** were consistent with those reported in the literature,^21^ with no chromatographic purification required this time.^21^ This high-yield outcome validates our hypothesis that a lower yield of **2** was due to the presence of hotspots in the reaction system. By avoiding chromatography, the whole production process was further speeded up.

**Table 1.** Effects of the different experimental parameters on the yield of intermediate **2**.

Method used for scale-up	Temp.	Yield of **2**	Purification	Time needed
This work	−10°C	93%	No	4 h
Literature^21^	4°C	50%^a^	Yes	2 days

a Scale-up of alcohol **2** from ester **1** using a literature protocol, 71% on a 200 mg scale,^20^ afforded **2** only in 50% yield.

After the successful improvement of the synthesis of intermediate **2**, a two-step, one-pot reaction was performed to obtain carbonate **3** from alcohol **2.** The efficiency of this reaction was improved by performing three modifications. First, *in-situ* formation of intermediate **4** from **2** was performed in CH_2_Cl_2_, followed by workup with brine. The use of a CH_2_Cl_2_/brine system allowed an easier removal of the reaction byproducts from intermediate **4**. No emulsions were observed and the migration of **4** in the aqueous phase (TLC analysis) during workup was greatly minimized, by contrast to what observed using the THF/water system.^22^ This evidence supports our hypothesis that a partial loss of intermediate **4** during workup was accountable for the low overall yield of **3**. Second, the formation of **3** from **4** was better accomplished by adding a CH_2_Cl_2_ solution of PNP chloroformate to a mixture of **4**, pyridine and CH_2_Cl_2_ at 0°C. This strategy eliminated the presence of hotspots and allowed a better temperature control of the system.

Finally, purification over deactivated silica minimized the decomposition of **3**, which was obtained in a better recovery with an overall yield of 60%. Given the negative environmental impact of performing chromatography of **3** on a large scale, we originally investigated greener, silica-free purification techniques (distillation and recrystallization). However, these techniques proved inapplicable given the physical nature of **3** (oil) and its known thermal instability.^21^ ^1^H NMR data of **3** were in agreement with the ones reported in the literature.^22^ A comparison between the two different experimental protocols are illustrated in the tables below. Tables 1 and 2 illustrate the improvements of the single reactions leading to alcohol **2** and carbonate **3**. Table 3 illustrates the difference of the two overall synthetic protocols on the total yield of **3**.

**Table 2.** Effects of the different experimental parameters on the yield of carbonate **3**.

Method used for scale-up	Temp.	Yield of **3**	Solvent	PNP-Cl added	Purification	Time needed
This work	0°C	65%	CH_2_Cl_2_	Solution in CH_2_Cl_2_ (over 1 h)	Deactivated silica	1 day
Literature^24^	0 ºC	10%^a^	THF	Solid (neat)	Normal silica	2 days

a Scale-up of carbonate 3 from alcohol 2 using a literature protocol, (3% on a 200 mg scale,23 afforded 3 only in 10% yield.

**Table 3.** Comparison between overall yields of carbonate **3**. Finally, our approach is highly reproducible. As a proof, we repeated the overall synthesis of **3** three times, obtaining **3** with the same degree of purity (94–95%). Yields were 60%, 58%, and 62% for repetitions 1–3, respectively.

Method used for scale-up	Overall yield of **3**	Solvent	Overall time needed
This work	60%	CH_2_Cl_2_	1 day and 4 h
Literature^20,23^	5%	THF	4 days

## Conclusions

We successfully developed an improved synthesis of a cyclopropene carbonate **3** in an unprecedented six-grams scale. Our approach has significant advances over the synthetic protocols currently available in the literature, both in terms of increased overall yield (60% versus 5%), reduced times (1 day and 4 h compared to 4 days), and minimized chromatographic purifications. Copper-free click chemistry has proven a highly exquisite and versatile approach to prepare a wide range of novel biomaterials and nanocomposites for biomedical and environmental applications. However, the applicability of this approach still suffers from a lack of feasible and cost-effective methods to prepare the required key chemicals in a larger scale. Our improved protocol can therefore fill this gap and encourage further research in the field of the copper-free click chemistry, with the long-term goal to promote this approach in the large production and commercialization of nanomedicine, contrast reagents, scaffolds and implantable devices for targeted drug delivery, clinical imaging, surgical reconstruction, ophthalmology, and environmental applications.
